# Association of iPACK block and adductor canal block in dogs undergoing the tibial plateau leveling osteotomy technique: Report of 4 cases

**DOI:** 10.29374/2527-2179.bjvm002324

**Published:** 2024-06-05

**Authors:** Desirée Santos da Rosa, Isabele de Matos Oliveira, Laryssa Reginaldo Ribeiro da Silva, Yasmin Santos Kaulich de Souza, Gustavo Nunes de Santana Castro

**Affiliations:** 1 Undergraduate in Veterinary Medicine, Universidade Iguaçu (UNIG), Nova Iguaçu, RJ, Brazil.; 2 Veterinarian, MSc., autonomous, Paracambi, RJ, Brazil; 3 Veterinarian, DSc. Programa de Pós-Graduação em Ciências Veterinárias , Departamento de Parasitologia Animal. Instituto de Veterinária, Universidade Federal Rural do Rio de Janeiro. Seropédica, RJ. Brazil.

**Keywords:** popliteal plexus, hunter channel, analgesia, guided block, popliteal plexus, hunter channel, analgesia, guided block

## Abstract

Orthopedic procedures are associated with severe postoperative pain. In TPLO, the block commonly used is the sciatic nerve block associated with the femoral nerve block. In orthopedic surgeries in human medicine, the iPACK block associated with the adductor canal block has been used as alternatives that do not affect the strength of the quadriceps femoris muscle. The objective of this study was to evaluate the trans and postoperative analgesic effect of the association of iPACK block and adductor canal block, as well as to evaluate the patient's motor recovery after surgery. Four patients were selected, without distinction of breed and gender, weighing more than 22lb, referred to TPLO. All patients underwent the combination of iPACK block and adductor canal block with 0.5% bupivacaine. The intraoperative evaluation was carried out by measuring mean arterial pressure, heart rate and respiratory rate, and all patients were stable during the procedure. The postoperative evaluation was carried out based on the assessment of pain using the modified Glasgow scale, in which all patients scored less than 05/24, and assessment of ambulation through videos using the adapted Muzzi scale, presenting ambulation between grade 1 and 2. No patient required intraoperative or postoperative analgesic rescue.

## Resumo

Os procedimentos ortopédicos estão associados a dores pós-operatórias severas. Em TPLO, o bloqueio comumente utilizado é o bloqueio do nervo ciático associado ao bloqueio do nervo femoral. Nas cirurgias ortopédicas em medicina humana, o bloqueio iPACK associado ao bloqueio do canal dos adutores tem sido utilizado como alternativa que não afeta a força do músculo quadríceps femoral. O objetivo deste estudo foi avaliar o efeito analgésico trans e pós-operatório da associação do bloqueio iPACK e bloqueio do canal adutor, bem como avaliar a recuperação motora do paciente após a cirurgia. Foram selecionados quatro pacientes, sem distinção de raça e sexo, com peso superior a 10kg, e encaminhados ao TPLO. Todos os pacientes foram submetidos a uma combinação de bloqueio iPACK e bloqueio do canal dos adutores com bupivacaína a 0,5%. A avaliação intraoperatória foi realizada através da aferição da pressão arterial média, frequência cardíaca e frequência respiratória, e todos os pacientes permaneceram estáveis durante o procedimento. A avaliação pós-operatória foi realizada com base na avaliação da dor pela escala de Glasgow modificada, na qual todos os pacientes pontuaram menos que 24/05, e avaliação da deambulação por meio de vídeos pela escala de Muzzi adaptada, mostrando deambulação entre grau 1 e 2. Nenhum paciente necessitou de resgate trans ou pós-operatório.

## Introduction

Insufficiency of the cranial cruciate ligament is the main condition of the hind limb in dogs, wich is almost always concomitant with other changes – such as lateral or medial patellar dislocation (LPL or LPM) (Chierichetti & Pedro, 2009). Despite being diagnosed in animals of different breeds and ages, it is most commonly observed in large and/or overweight dogs, such as Labradors, Rottweillers and Staffordshire Terriers (Spinella et al., 2021). Orthopedic surgeries are associated with moderate to severe pain (Ekman & Koman, 2004), making it extremely important to consider perioperative analgesia. To achieve this, the anesthetist uses multimodal analgesia techniques that include pre-anesthetic medication and locoregional blocks, helping to reduce or eliminate the transmission of the painful stimulus in a reversible manner (Reschke & Gorczack, 2022), which is important for stabilization of the patient during surgery and better postoperative recovery.

The most commonly used locoregional block for Tibial Plateau Leveling Osteotomy (TPLO) is the association between sciatic nerve block and femoral nerve block. However, they present some important effects in the postoperative period in human patients, such as loss of muscle tone (Zheng et al., 2022). In dogs, there is also a loss of muscle tone, since the femoral nerve and the sciatic nerve are also responsible for the muscle tone of the pelvic limb (Evans & De Lahunta, 2013; Otero & Portela, 2021). The femoral and sciatic nerves are the main branches of the lumbosacral plexus, and the combined block of these two nerves results in anesthesia of the pelvic limb almost in its entirety (Campoy et al., 2008), desensitizing the final third of the femur, knee, tibia, tarsus and digits (Mahler & Adogwa, 2008). Although it achieves a satisfactory analgesic effect, it has its results in quadriceps femoris and calf weakness, which limit early autonomous exercise and joint activity and increase the risk of falls after surgery (Zheng et al., 2022). iPACK block and adductor canal block are alternatives for intraoperative analgesia used in human orthopedic surgeries for total knee arthroplasty (TKA) which have shown good analgesic efficacy with fewer postoperative effects (Sinha et al., 2022), promoting faster patient recovery.

The interspace between the popliteal artery and the posterior capsule of the knee (iPACK) block consists of infiltrating local anesthetic into the interspace between the popliteal artery and the posterior knee capsule, inserting the needle into the posterior aspect of the popliteal artery and targeting the popliteal plexus (Sonawane et al., 2021). iPACK blocks the terminal branches of the genicular nerves and popliteal plexus, responsible for most of the sensory innervation of the posterior capsule of the knee joint (Et et al., 2022). The objective is to selectively block only the innervation of the posterior knee joint, sparing the main trunks of the tibial and common peroneal nerves – branches of the sciatic nerve that provide motor and sensory innervation –, thus maintaining the sensory-motor function of the leg (Moreira et al., 2016, Et et al., 2022). Adductor canal blockade, in human medicine, is considered an element of multimodal analgesia in patients undergoing total knee arthroplasty due to its opioid savings and motor protective effects (Et et al., 2022).

Associated with iPACK, the adductor canal block is used, the needle is inserted at the mid-thigh level, advancing to the femoral artery, targeting the saphenous nerve – a nerve considered purely sensory – and, indirectly, the popliteal plexus through dispersion (Bowley & Doughty, 2018; Sonawane et al., 2021). By combining both blocks, it generates satisfactory analgesia, without affecting the strength of the quadriceps femoris muscle.

The purpose of the present work is to study a technique used in humans, which has good efficacy and which, if confirmed in animals, can be used routinely for pelvic limb surgeries involving the knee joint. This results in a quality post-operative period, rapid return to activities, shorter hospital stays and rest, which is also a necessity for owners, as it makes the animal's post-operative management less complicated.

## Cases report

For the present work, 4 dogs were selected, without distinction of breed and gender, weighing at least 22lb, sent for tibial plateau leveling osteotomy surgery (TPLO). The inclusion criteria were: docile and healthy animals. The exclusion criteria were: animals under 22lb; animals with concomitant diseases of cranial cruciate ligament insuffiency (CrCLI); and/or animals showing clinical and laboratory changes. The Ethics Committee for the Use of Animals (CEUA) at Universidade Iguaçu approved the research project.

Clinical examinations were performed, including: anamnesis, evaluation of parameters such as heart rate, respiratory rate, among other important pre-surgical criteria for the anesthetic protocol, in addition to routine pre-surgical exams, such as blood count and measurement of kidney and liver enzymes.

Dogs were premedicated with Acepromazine (0.03 mg/kg IM) and Meperidine (3 mg/kg IM). After 30 minutes, the cephalic vein was punctured for venous access. Fluid therapy with Ringer's lactate was started at a rate of 5ml/kg/hr. Anesthetic induction was performed with propofol at a rate of 2 mg/kg/min until the absence of eyelid and laryngeal reflexes. At that moment, the patients were intubated, starting the administration of isoflurane with 100% oxygen. Heart rate, respiratory rate, blood pressure and oxygen saturation were measured during the anesthetic procedure and, if there was a variation of 20% or more in the first three parameters, a continuous infusion of remifentanil would be initiated for analgesic management during the intraoperative period at a rate of 0.5 mcg/kg/hr.

**Figure 1:**
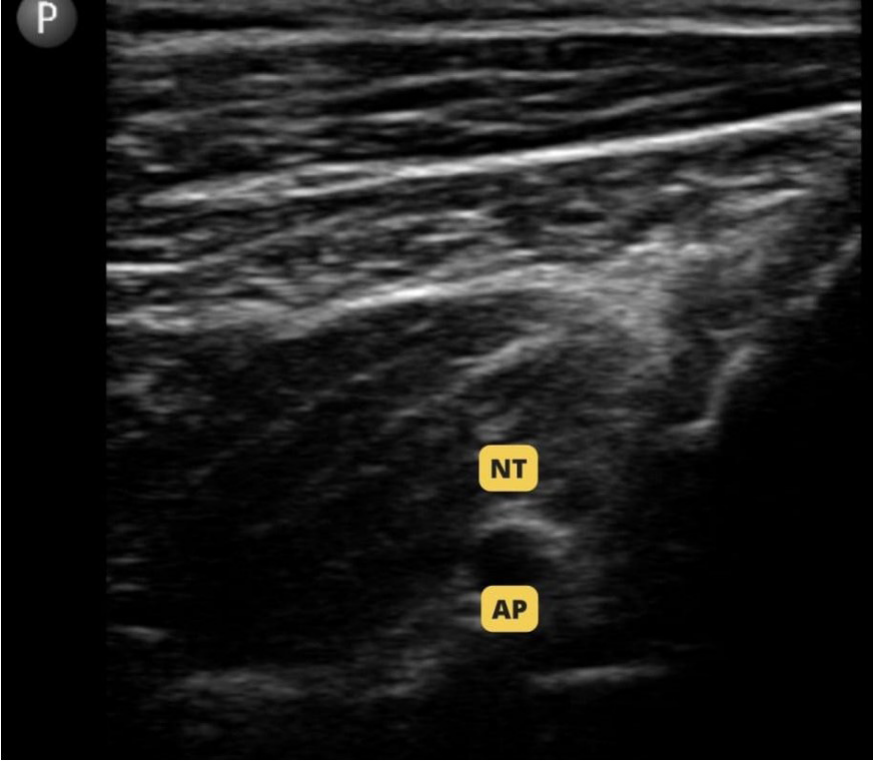
Ultrasound scan of the iPACK lock. AP: popliteal arteries; NT: tibial nerve.

For the iPACK block, the pelvic limb was trichotomized and antisepsis was performed with 2% Chlorhexidine Digluconate and 0.5% alcoholic Chlorhexidine Digluconate. Ultrasonography was scanned until the popliteal artery was visualized (Figure 1). At that moment, the needle from a 20G catheter was introduced, with the syringe containing 2 mL of 0.5% Bupivacaine (0.05 mL/kg). The anesthetic was injected after the negative pressure test, targeting the interspace between the popliteal artery and the posterior capsule of the knee. During this block, it was observed on ultrasound that there was not enough window to advance the needle. Therefore, the anesthetic was introduced a little before the indicated location, so it could reach the nerve by dispersion.

**Figure 2:**
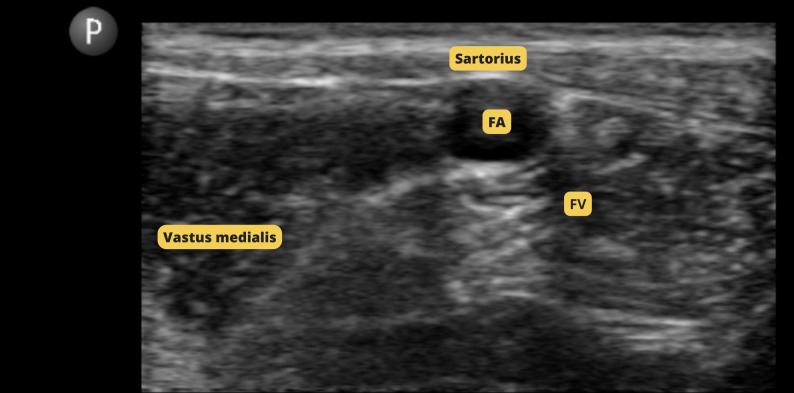
Ultrasound scan of Adductor Canal blockage. AF: femoral artery; VF: femoral vein.

To block the adductor canal, the aseptic technique was performed and ultrasound scanning was performed until the femoral artery was visualized (Figure 2). At that moment, the needle was introduced, with the syringe containing 2 mL of 0.5% Bupivacaine (0.05 mL/kg). Aspiration was performed for the negative pressure test and, subsequently, the anesthetic was introduced targeting the saphenous nerve.

The analysis was carried out intraoperatively, by evaluating the consumption of remifentanil for analgesic management, and post-surgical assessment of recovery and pain levels, in addition to post-surgical morphine consumption. Post-surgical pain was measured using the Glasgow Scale (Reid et al., 2007), and if the patient had a score equal to or greater than 05, they would receive 0.2 mg/kg of morphine, with an assessment being carried out every 15 minutes. Patients were only released with a score below 05, at which point they received Meloxicam (0.2mg/kg subcutaneously) and Dipyrone (25mg/kg subcutaneously). Furthermore, patients' ambulation was assessed using the adapted Muzzi scale (Muzzi et al., 2003). The information on intraoperative parameters can be seen in Table 1.

**Table 1:** Mean intraoperative parameters.

***Mean intraoperative parameters***				
	*Patient 1*	*Patient 2*	*Patient 3*	*Patient 4*
Heart rate	100 bpm	110 bpm	90 bpm	100 bpm
Mean arterial pressure	90 mmHg	100 mmHg	70 mmHg	80 mmHg
Respiratory rate	20 rpm	30 rpm	20 rpm	20 rpm

Note: This table shows the mean intraoperative parameters of the 4 patients during the procedure.

The first patient was a 1-year-old Rottweiler, 90 lb, body condition score 4, with right limb affected. The patient presented mild sedation, no vomiting and no excitement. The patient remained stable throughout the procedure, with no major changes in the main variables. The heart rate (HR) remained at 100 bpm during most of the procedure, with the highest HR being 120 bpm and the lowest being 90 bpm. Mean arterial pressure also remained stable during the procedure, around 90 mmHg. The respiratory rate also remained stable, at 20 rpm throughout the procedure, with only a peak of 30 rpm. At postoperative evaluation, the pain score was 2, 2, 2, so the patient did not require morphine rescue. Walking was assessed 4 hours after the surgical procedure, using videos sent by the tutor, and was defined as grade 2 (mild lameness only after exercise or prolonged recumbency).

The second patient was a 3-year-old Golden Retriever, 94 lb, body condition score 4, with right limb affected. The patient presented moderate sedation, no vomiting and no excitement. The HR remained at 110 bpm for most of the procedure, however, it underwent changes. It did not exceed the recommended 20% change and did not require analgesic rescue. The RR remained at 30 rpm, and the MAP also remained stable (100 mmHg), presenting a peak of 130 mmHg at a given moment during the surgery, but stabilizing 10 minutes later. In the postoperative evaluation, the pain score was 2, 2, 1, with no need for morphine rescue. Ambulation was assessed 4 hours after the procedure, classified as grade 1 (Absent lameness, complete support of the limb with the animal in a station or during physical activity).

The third patient was a 9-month-old Pitbull, 57 lb, body condition score 3/5, with left limb affected. The patient presented mild sedation, no vomiting and no excitement. The HR remained between 80 and 90 bpm during much of the procedure. The RR remained at 20 rpm. MAP presented a peak of 120 mmHg, however, it was a matter of the anesthetic plane, in which the patient appeared more superficial in relation to the Guedel plane. During the entire procedure, it remained between 60 and 70 mmHg. Remifentanil analgesic rescue was not necessary intraoperatively. The postoperative pain assessment showed scores of 3, 3 and 2, meaning that analgesic rescue was not necessary. Walking was assessed 4 hours after the procedure, classified as grade 2 (mild lameness only after exercise or prolonged recumbency).

The fourth patient was a 3-year-old Chow Chow, 59 lb, body condition score 3, with right limb affected. The patient presented moderate sedation, without vomiting or excitement. HR remained between 95 and 100 bpm during the procedure, with a peak of 110 bpm 5 minutes before the end of the surgery. The RR remained at 20 rpm. MAP remained between 65 and 80 mmHg, with a peak of 105 mmHg 30 minutes after the start of the procedure. Intraoperative analgesic rescue was not necessary. During the postoperative pain assessment, the patient scored 3, 2 and 2 in the three assessments performed, also not requiring morphine analgesic rescue in the postoperative period. Walking was assessed 4 hours after the procedure, classified as grade 2 (mild lameness only after exercise or prolonged recumbency).

## Discussion

Cranial cruciate ligament insufficiency can occur in animals of any breed, size and age, however, it is more common in larger and younger dogs (Mateus, 2010). In large and/or overwheight dogs, the cranial cruciate ligaments tends to suffer greater stress, which accelerates degeneration, predisposing to rupture (Vasseur, 1985; Spinella et al., 2021), increasing the chance of developing the disease and explaining why it occurs more commonly than in small dogs. Furthermore, Vasseur (2002) states that dogs under 4 years of age present traumatic rupture more easily, unlike dogs aged from 5 to 7 years, which present the disease caused by degeneration that leads to spontaneous rupture. The selected patients were Pitbull, Golden Retriever and Chow Chow breeds, which is in line with the information provided by the two authors.

Meperidine is an opioid with 10% of the analgesic potency of morphine, with a half-life of 3 hours (Gozzani, 1994), and which also has sedative potential (Botan & Lapena, 2015). As a complement to the protocol, acepromazine was used, a tranquilizing derivative of phenothiazine, which does not have analgesic efficacy, but potentiates the analgesic effects of opioids (Yamazaki et al., 2011). Once the objective was to evaluate the analgesic potential of the block, it was decided to use a pre-anesthetic protocol using a weak opioid, so it did not produce such intense analgesia and it was possible to evaluate the analgesia of locoregional blocks.

One patient presented tachycardia at one point during surgery, but without presenting the possible causes previously mentioned. Furthermore, Sousa et al. (2022) states that the association of Acepromazine and Meperidine does not cause major cardiovascular changes. Therefore, it was understood that the increase in heart rate was correlated with inadequate anesthetic depth. Along with this, hypertension can also occur due to the aforementioned causes, however, in the patient in question, the anesthetic depth was assessed using the Guedel plane and superficialization to stage 3, plane 1 was noted, which would explain the changes seen and not requiring intraoperative analgesic rescue. After the plan adjustment, the parameters stabilized.

To this purpose, the use of locoregional anesthesia is discussed, which prevents nociceptive input to the central nervous system, promoting transoperative analgesia and greater postoperative comfort. Sankineani et al. (2018) carried out a study with 120 patients undergoing total knee arthroplasty – 60 patients received the Adductor Canal block alone, and 60 received the iPACK block associated with the Adductor Canal –, and patients in the iPACK + Adductor Canal group showed better pain scores, greater range of motion and greater walking distance 8 hours after the procedure. In fact, the patients in this study had low pain scores (Modified Glasgow scale) and good ambulation scores (adapted Muzzi scale).

Zheng et al. (2022) evaluated the impact of iPACK combined with Adductor Canal block on early motor function after TKA, comparing with sciatic nerve block associated with femoral nerve block, and observed that iPACK + Adductor Canal had little impact on muscular strength and with a superior analgesic effect compared to commonly performed blocks. In human medicine, these results are expected so that the patient can perform activities outside of bed and start early physical therapy. In veterinary medicine, such results demonstrate a lower risk of falling by the patient – with a lower chance of injury to the affected limb or the contralateral limb –, in addition to facilitating the post-operative period for owners.

Abdullah et al. (2022) compared the iPACK block associated with the Adductor Canal block and the Adductor Canal block alone, evaluating postoperative analgesia and walking ability in human patients undergoing TKA. As a result, they observed that the group that received the two combined blocks had lower pain scores and lower morphine consumption in the postoperative period 4, 6 and 12 hours after the procedure, finding no significant difference in the ability to walk. This result is partially in line with this work, since the patients actually had low pain scores, not requiring morphine analgesic rescue in the 3 pain assessments in the immediate postoperative period. However, good ambulation scores were also observed, in which patients were able to walk without limping shortly after surgery.

Beckhauser et al. (2019) reported a human patient undergoing TKA who received the iPACK block associated with the Adductor Canal block. In their report, they observed that the patient had preserved motor strength during physiotherapy on the same day of the procedure, with no pain at rest and pain during movement of 5/10. Morphine was prescribed as rescue analgesia, however, its use was not necessary. The results are in line with the patients in the present study, as they presented complete or partial support of the limb, without lameness, 4 hours after the surgical procedure, a sign of preserved motor strength. Furthermore, analgesic rescue was not necessary during the intraoperative or postoperative period in any of these cases.

Et et al. (2022) evaluated 105 human patients undergoing TKA, who were divided into 3 groups: iPACK + Adductor Canal, Periarticular Infiltration + Adductor Canal, and Adductor Canal alone. They then observed that the group in which the iPACK block was added had a shorter hospital stay, faster movement, in addition to improving pain scores in the 48 postoperative hours and, consequently, reducing the consumption of opioids.

Sinha et al. (2022) evaluated the analgesic effect of continuous adductor canal block compared to femoral nerve block in human patients undergoing arthroscopic knee surgery, and observed that cumulative analgesic consumption 24 hours after the procedure was lower in the adductor canal group. There was also a significant difference in the range of painless movement, with the adductor canal group being superior in terms of analgesia and preservation of quadriceps femoris muscle strength.

There are no studies published in Veterinary Medicine reporting the use of the blocks mentioned in this work, and there are also no studies comparing them with the commonly used blocks (Sciatic + Femoral). Therefore, studies carried out in human medicine were used to support the information and results of this work.

## Conclusions

The results of the present study demonstrated that the iPACK block associated with adductor canal block seem to be good options to replace sciatic nerve blocks associated with femoral nerve block in TPLO surgeries. These techniques can reduce the animals' hospitalization time, improve early mobility, and lower the risk of falls by preserving the strength of the quadriceps femoris muscle, in addition to reducing the consumption of opioids intraoperatively and postoperatively. However, studies with a larger N of patients evaluated are needed.
